# Optimization of critical parameters for coating of polymeric nanoparticles with plasma membrane vesicles by sonication

**DOI:** 10.1038/s41598-021-03422-5

**Published:** 2021-12-14

**Authors:** Feipeng Yang, Maleen H. Cabe, Sean D. Ogle, Veronica Sanchez, Kelly A. Langert

**Affiliations:** 1 Department of Molecular Pharmacology and Neuroscience, Loyola University Chicago, Stritch School of Medicine, Maywood, IL 60153 USA; 2 Research Service, Edward Hines Jr. VA Hospital, Hines, IL 60141 USA

**Keywords:** Drug delivery, Nanoparticles, Membrane structure and assembly

## Abstract

Top-down functionalization of nanoparticles with cellular membranes imparts nanoparticles with enhanced bio-interfacing capabilities. Initial methods for membrane coating involved physical co-extrusion of nanoparticles and membrane vesicles through a porous membrane; however, recent works employ sonication as the disruptive force to reform membranes around the surface of nanoparticles. Although sonication is widely used, there remains a paucity of information on the effects of sonication variables on coating efficiency, leading to inconsistent membrane coating across studies. In this work, we present a systematic analysis of the sonication parameters that influence the membrane coating. The results showed that sonication amplitude, time, temperature, membrane ratio, sample volume, and density need to be considered in order to optimize membrane coating of polymeric nanoparticles.

## Introduction

Top-down functionalization of nanoparticles with cell membranes imparts several key properties to particles that facilitate systemic administration and targeted drug delivery^1^. Unlike coating with biocompatible hydrophilic polymers (e.g. polyethylene glycol) to achieve increased systemic circulation, coating with cellular membranes allows for enhanced avoidance of the host immune response^2,3^. In addition, retained transmembrane proteins enable active targeting to inflamed or diseased tissue^4,5^. For these reasons, cell membrane cloaked, or biomimetic, nanoparticles have seen an influx of recent interest as a targeted drug delivery system^6^.

Several methods can be used to fuse membrane material onto nanoparticle cores, including extrusion^7,8^, sonication^9–11^, electroporation^12^, and microfluidic platforms^13^. Of these methods, extrusion and sonication are most frequently used. When methods such as electroporation or microfluidics are employed, sonication and/or extrusion are still necessary for top-down preparation of unilamellar membrane vesicles from source cells^12,13^. Microfluidic devices facilitate streamlined mixing of membrane vesicles and NP cores; however, exogenous force in the form of sonication^13^ or electroporation^12^ is required for complete coating of NP cores. Thus, despite advances in alternative methods for membrane coating onto NPs, extrusion and sonication remain the most frequently used.

Extrusion and sonication employ physical principles (shear force and acoustic force, respectively) to either deform or rupture membrane vesicles and allow them to reassemble around cores in an energetically favorable process^14^. Advantages of extrusion include a high degree of control and reproducibility, due to the syringe-based construction of commercial extruder devices. Samples obtained with extrusion are free from contaminants that are greater in size than the well-defined pores, and they are monodisperse in size with high batch-to-batch reproducibility^15,16^. Advantages of sonication include higher yield and no material lost as syringe dead volume, which is in stark contrast to the low throughput nature of extrusion^6,17^. We argue that with careful optimization of variables to control for reproducibility, the advantages of sonication outweigh the disadvantages, as a low yield is prohibitive for functional assays and in vivo studies.

The disruptive forces generated by sonication lead to the spontaneous fusion of nanoparticles with membrane vesicles to form a core–shell nanostructure^3^. As sonication utilizes acoustic energy, several variables influence the interactions between nanoparticles and membrane vesicles in co-suspension^18,19^. These variables include the standardly reported power, amplitude, and duration of sonication, but also the density of particles and membrane vesicles in the suspension, the volume of the suspension, and the temperature of the bath. We have previously found (data not shown) that neglecting to optimize and control for each of these parameters and their interactions results in inconsistent membrane coating efficiency. Complete removal of unconjugated material is difficult to achieve, and methods to do so result in decreased yield. These issues compound to make it difficult to achieve reproducibility in functional or in vivo assays, particularly as interest in biomimetic delivery systems continues to grow.

Here, we present a systematic analysis of the parameters that influence the behavior of co-suspensions of polymeric nanoparticles and membrane vesicles. By gaining a more thorough understanding of the variables that influence the completeness and efficiency of membrane coating, experiments can be better designed, and results can be more easily reproduced by other groups and translated to clinical applications.

## Results

**Figure 1:**
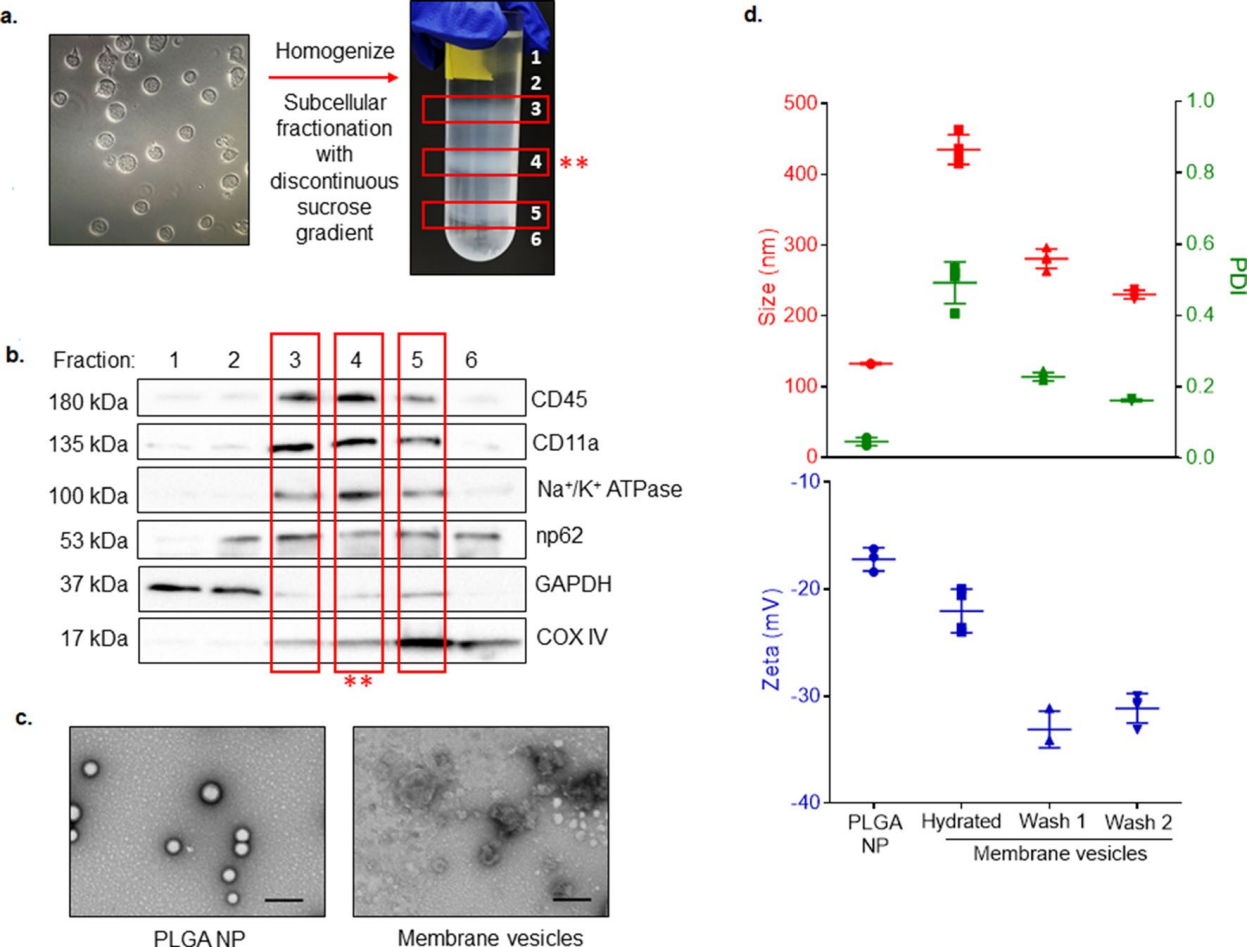
Schematic of membrane vesicle and PLGA nanoparticle (NP) preparation. (**a**) Images of Jurkat T cells (scale bar: 100 µm) and of cell homogenate separated into three lipid-rich subcellular fractions after density gradient centrifugation. The fraction from the 30–40% interface (red asterisks) was used for downstream experiments. (**b**) Western immunoblot demonstrating the protein profile of six collected subcellular fractions. Antibodies to plasma membrane (CD45, CD11a, Na^+^/K^+^ ATPase), nuclear membrane (nucleoporin 62 (np62)), mitochondrial membrane (cytochrome C oxidase subunit 4 (COXIV)), and cytosolic GAPDH were used. (**c**) Transmission electron microscopy images depicting morphology of PLGA NP cores and rehydrated membrane vesicles. Scale bar: 200 nm. (**d**) Size (nm, red), polydispersity index (PDI, green), and zeta potential (mV, blue) of PLGA NPs and of membrane vesicles at each stage of processing. Data are the mean ± SD, *n* = 3–4 separate preparations.

Subcellular fractions were isolated from Jurkat T lymphocytes as previously described^20^. After separation of fractions with a discontinuous sucrose gradient (Fig. 1a), six separate fractions were collected and probed for key membrane proteins (CD11b, CD45, and sodium–potassium ATPase (Na^+^/K^+^ ATPase; all plasma membrane), nucleoporin p62 (nucleus), and cytochrome c oxidase (COX IV, mitochondria) and for cytosolic GAPDH using a Western immunoblot. The lipid-rich fraction collected from the 30%/40% interface demonstrated abundant plasma membrane markers (CD11b, CD45, Na^+^/K^+^ ATPase) and minimal nuclear (np62) and mitochondrial (COXIV) markers, indicating that this fraction is enriched with plasma membrane vesicles (Fig. 1b). Uncropped blots are included as Supplementary Fig. S1. Upon reconstituting lyophilized fraction with deionized water (diH_2_O), lipids form large, polydisperse vesicles (435.1 ± 20.92 nm, PDI: 0.49 ± 0.058). We performed a series of vortexing, washing, and sonicating to remove residual salts and impurities, resulting in a monodisperse population (PDI 0.1615 ± 0.003) with an average diameter of 230.3 ± 6.138 (Fig. 1c, d). The zeta potential of plasma membrane vesicles shifted from  − 22.03 ± 2.048 mV to  − 31.14 ± 1.374 mV after the two wash steps.

PLGA nanoparticles were formed by single emulsion with solvent evaporation and diffusion^21^. The inclusion of acetonitrile as a water-miscible co-solvent resulted in an average size of 132.7 ± 1.21 nm and a PDI of 0.046 ± 0.011 (Fig. 1c, d). A negative surface charge is necessary for unilamellar membrane coating, as positively charged particles have been shown to form large polydisperse aggregates when combined with membrane vesicles^22^. Further, the presence of extracellular glycans and proteins bestows a sidedness to membrane vesicles. Negatively charged NP cores facilitate outside-out re-assembly of membrane vesicles after sonication and contribute to the energetically favorable core–shell structure^22^. Our nanoparticles were negatively charged, with a zeta potential of -17.19 ± 1.09 mV.

### Effects of sonication temperature

**Figure 2:**
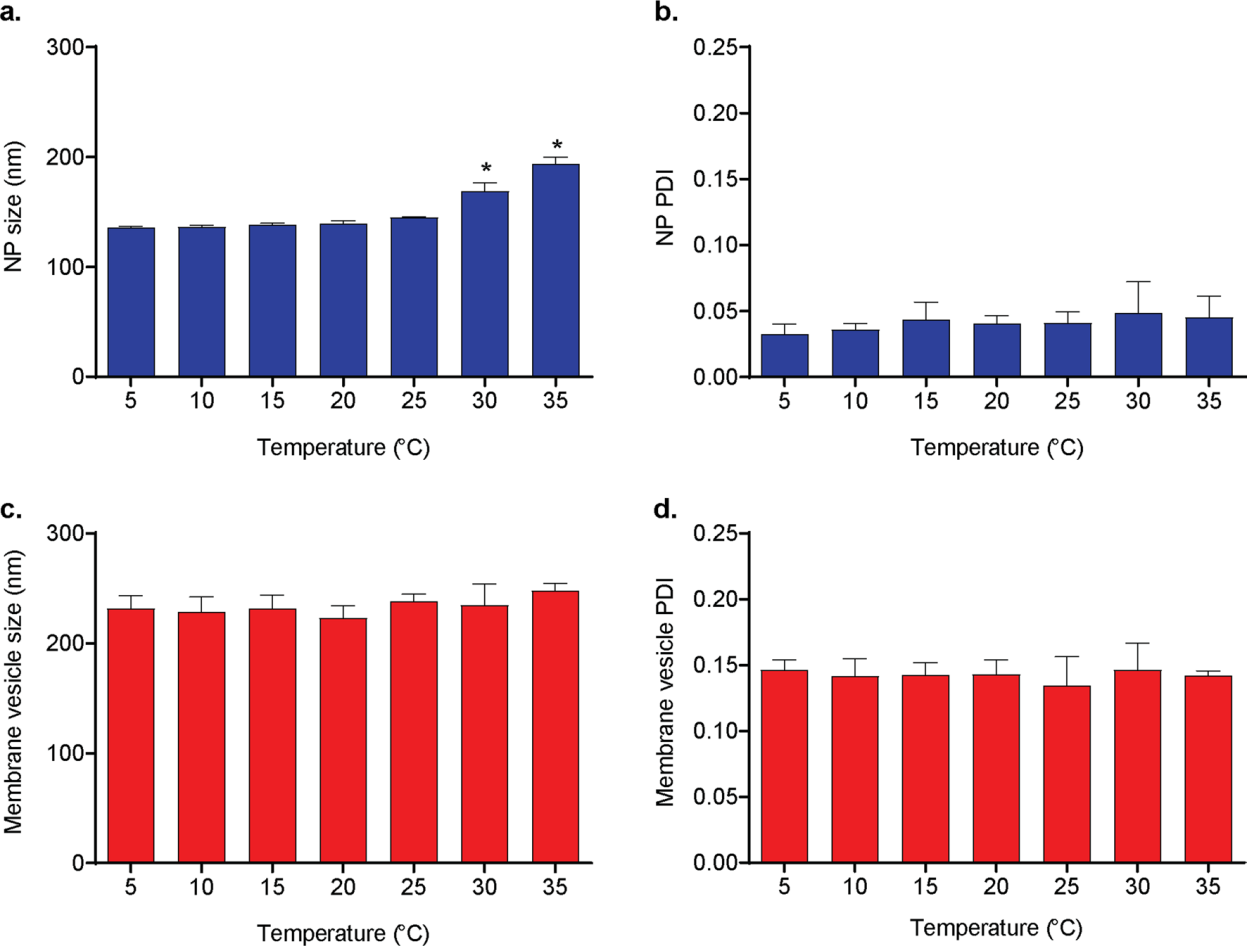
Effect of bath temperature on size and polydispersity of membrane vesicles and PLGA nanoparticles (NPs). (**a**) PLGA NP size (nm), (**b**) PLGA NP polydispersity index (PDI), (**c**) membrane vesicle size, and (**d**) membrane vesicle PDI after sonication at different temperatures for five minutes at 100% amplitude. For all datasets, shown are mean ± SD, *n* = 3 separate preparations. *p < 0.001 vs. all other temperatures, 1-way ANOVA with Tukey’s post hoc test.

The temperature of the water bath and the sample during bath sonication can affect both polymeric nanoparticle phase transition^23^ and membrane fluidity^24^. In the absence of external means of temperature control, the temperature of the bath increases during sonication^25^. To identify the optimal temperature of the bath as controlled by a recirculating chiller during sonication, nanoparticles (NPs, 0.5 mg/ml) were sonicated at 100% amplitude at a range of temperatures from 5 °C to 35 °C for 5 min. After sonication, samples were diluted with diH_2_O to a final NP concentration of 0.1 mg/ml, and size and PDI were evaluated with dynamic light scattering. NP size remained consistent after sonicating at 5–25 °C (range: 136.1–145.3 nm) and increased to 168.8 ± 7.87 nm and 194.0 ± 5.77 nm at 30 °C and 35 °C, respectively (Fig. 2a). The PDI did not change at any of the temperature settings (Fig. 2b), indicating uniform increases in size throughout the population.

Plasma membrane vesicles (0.1 mg/mL) were similarly sonicated at a range of temperatures from 5 °C to 35 °C and evaluated for changes in size and PDI after sonication. Vesicles did not exhibit any change in size or PDI at any of the temperature settings investigated (Fig. 2c, d).

### Assessment of coating efficiency by increases in size and PDI

**Figure 3:**
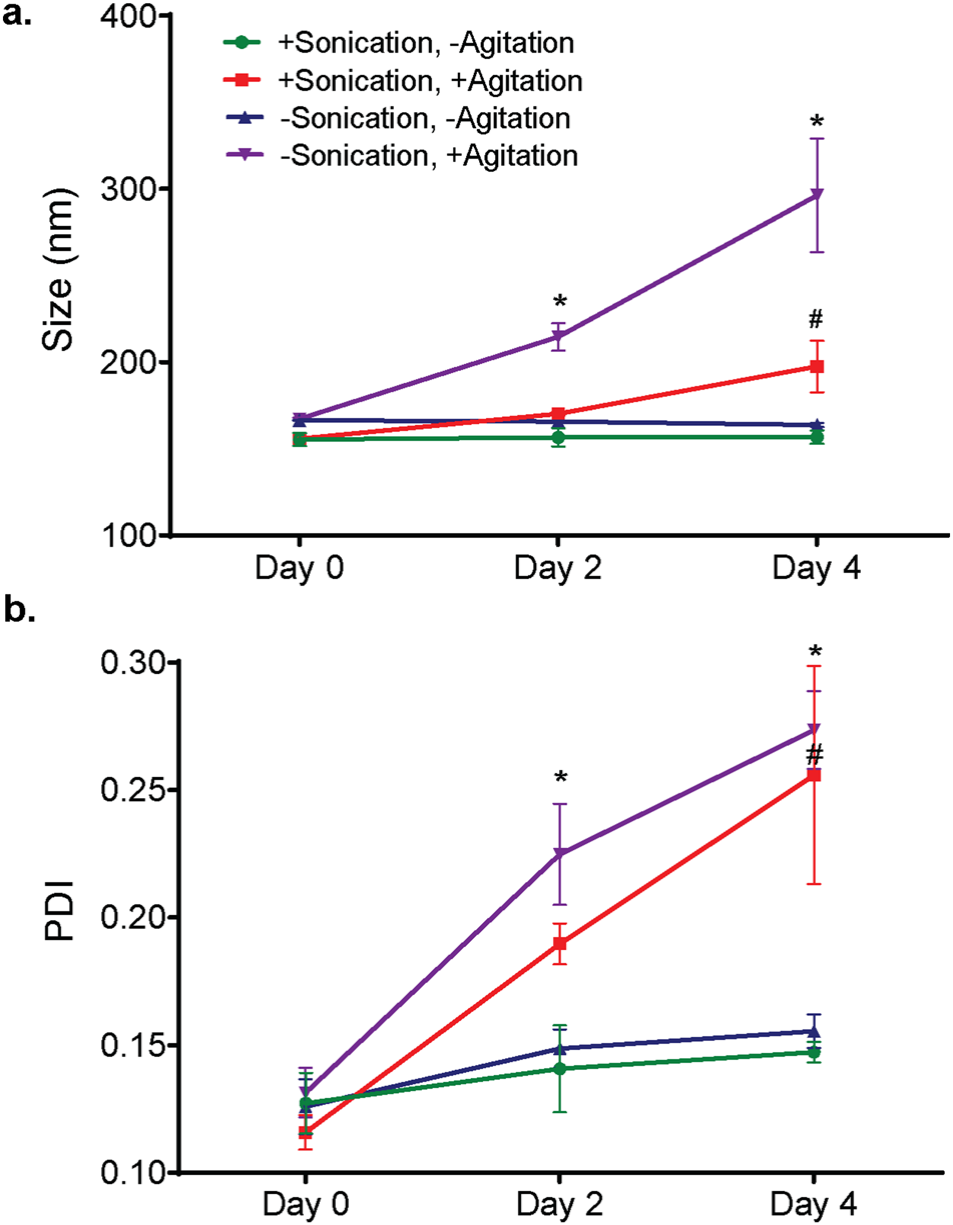
Effects of sonication and constant agitation on nanoparticle stability. Co-suspensions of PLGA NPs and membrane vesicles were sonicated (5 min., 15 °C, 60%) to induce membrane coating and then incubated at room temperature under static conditions (green line) or constant agitation (360° rotation at 8 rpm, red line). Control co-suspensions were not sonicated and then incubated as described above, with (purple line) or without (blue line) agitation. (**a**) Size (nm) and (**b**) polydispersity index (PDI) were assessed by dynamic light scattering on Day 0 (after sonicating), Day 2, and Day 4. Data shown are mean ± SD, *n* = 3. * *p* < 0.05, versus the other groups on the indicated day; # *p* < 0.05 versus Day 0. 2-way ANOVA with Tukey’s post hoc test.

To validate the quantitative outcomes of our experimental design, NPs were mixed with membrane vesicles in diH_2_O and sonicated (60% amplitude, 15 °C, 5 min.). After sonication, samples were diluted to a final NP concentration of 0.1 mg/ml in 1 × PBS and incubated at room temperature under constant agitation. Parallel samples were either not sonicated or not agitated. On day 0, there was no significant difference in size or PDI of the groups (Fig. 3a, b). On day 2, constant agitation began to promote aggregation of co-suspensions that were not sonicated to induce coating of NPs, as evident by a significant increase in size (to 214.6 ± 7.86 nm) and PDI (to 0.225 ± 0.02) compared to other groups. On day 2, agitated co-suspensions in which coating of available membrane vesicles onto NPs was induced by sonication exhibited a slight, significant increase in PDI only, from 0.116 ± 0.01 to 0.190 ± 0.008 (Fig. 3b).

On day 4, the average size and polydispersity of agitated co-suspensions in which the NPs were not coated with the membrane vesicles by sonication increased further, to 296.27 ± 32.8 nm with a PDI of 0.273 ± 0.015 (Fig. 3a, b). Agitated co-suspensions in which coating of available membrane vesicles onto NPs was induced by sonication increased in size from their Day 2 measurement to 197.5 ± 14.78 nm, and the PDI increased to 0.256 ± 0.043 (Fig. 3a, b). The average size of the co-suspension that was not sonicated was significantly greater (*p* < 0.001) than that of the co-suspension that was sonicated, indicating that coating with membrane vesicles did impart some stability to NPs.

Co-suspensions that were not incubated under constant agitation exhibited no change in size or polydispersity over four days, regardless of whether the NPs in the suspension were coated with available membrane vesicles by sonication (Fig. 3a, b, blue and green traces). These data indicate that constant agitation is necessary to reveal differences in coating efficiency, and that sonication induces coating of NPs with available membrane vesicles. Aggregation (increased size and PDI after 4 days of constant agitation) of NPs that were coated with available membrane vesicles suggests that NPs in the co-suspension were in excess. Next, we optimized the ratio of the two components of the co-suspension.

### Effect of membrane to PLGA NP ratio on coating efficiency

In an optimized experimental protocol utilizing membrane-coated NP cores, the ratio of each component will be empirically determined such that neither component is in excess. To find the optimal ratio of our PLGA NP cores and Jurkat plasma membrane vesicles, we investigated size as a function of membrane:NP weight ratio. NPs (0.7 mg/mL final concentration) were combined with different amounts of membrane vesicles in a final volume of 150 uL. The co-suspensions were sonicated at 20% amplitude, 5 min, 15 °C. After sonication, samples were diluted to a final NP concentration of 0.1 mg/mL in PBS and incubated at room temperature under constant agitation. Size and PDI were measured immediately after sonication (Day 0), on Day 2, and Day 4.

**Figure 4:**
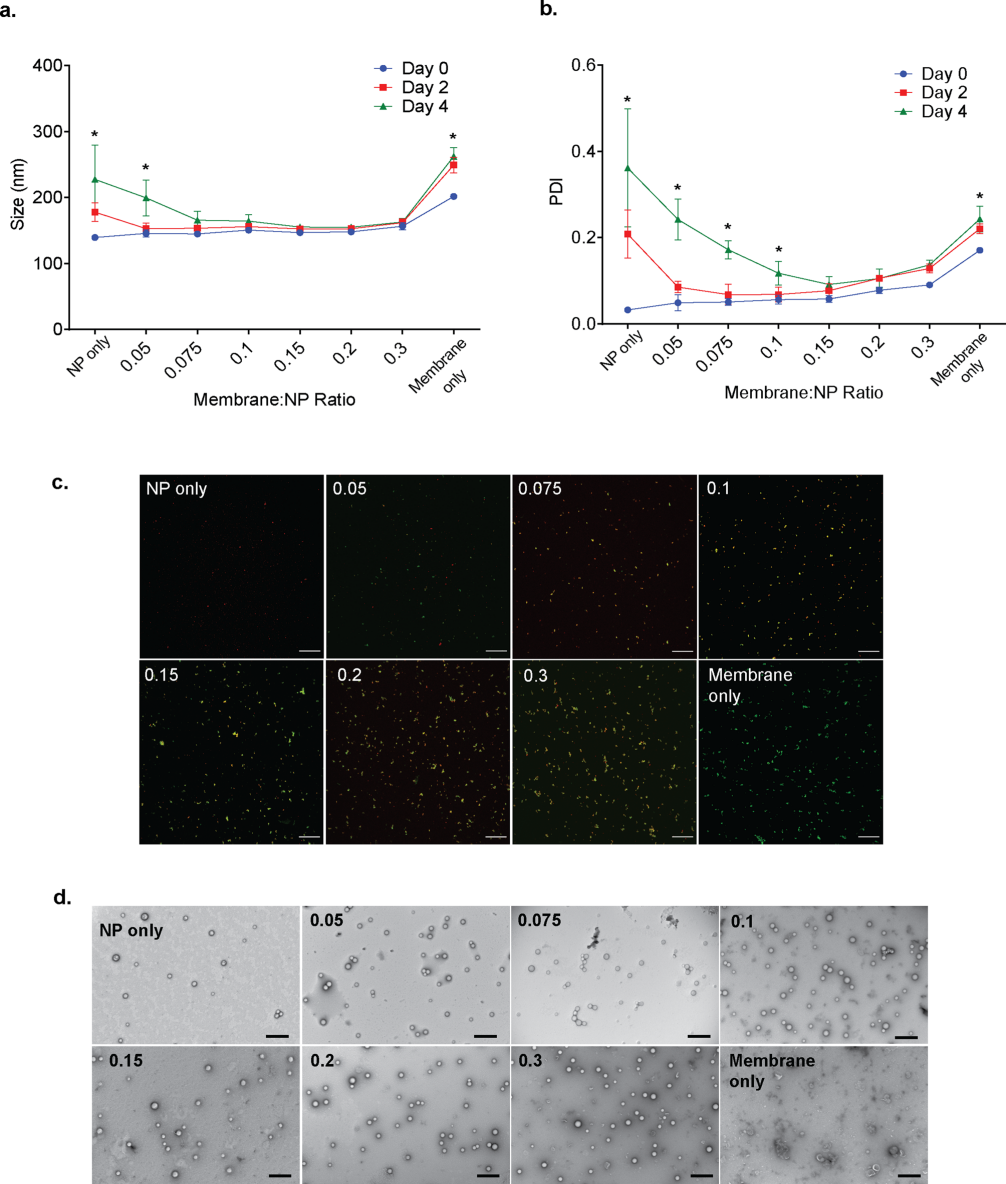
Effect of the ratio of membrane vesicles to nanoparticle cores on the efficiency of NP coating. Co-suspensions of different indicated ratios of membrane vesicles and PLGA nanoparticles (NPs) were sonicated (5 min., 15 °C, 60%) to induce membrane coating and then incubated at room temperature or under constant agitation (360° rotation at 8 rpm). (**a**) Size (nm) and (**b**) polydispersity index (PDI) were assessed by dynamic light scattering on Day 0 (after sonicating), Day 2, and Day 4. Data shown are the mean ± SD, n = 3. *p < 0.001 Day 0 versus Day 4 for the indicated ratios, 2-way repeated measures ANOVA with Dunnett’s post hoc test. (**c**) Representative confocal images depicting sonicated co-suspensions of indicated ratios of membrane vesicles (labeled with DiO, green) to NP cores (loaded with DiD, red) on Day 0. Yellow indicates co-localization of NPs and membrane vesicles. Images of data represented by the blue line in A and B. Scale bar: 20 μm. (**d**) Representative transmission electron microscopy images depicting sonicated co-suspensions of indicated ratios of vesicles and cores after 4 days of constant agitation (represented by the green line in A and B). Scale bar, 400 nm.

NPs alone in suspension exhibit significant increases in variability of size and PDI after two and four days of constant agitation (Fig. 4a, b). The addition of membrane vesicles at a membrane protein:NP weight ratio of 0.05 or 0.075 results in a size that trends downward after four days of constant agitation (Fig. 4a) but remains significantly increased over Day 0. The Day 4 PDI of these weight ratio groups (0.242, 0.172, respectively) are significantly higher (*p* < 0.05) than the Day 0 PDI (Fig. 4b). At a membrane protein:NP weight ratio of 0.15, there is no significant change in size (Fig. 4a) or PDI (Fig. 4b) at Day 2 or Day 4.

At membrane protein:NP weight ratios of 0.2 or greater, suspensions exhibit significant increases in size (Fig. 4a) and/or PDI (Fig. 4b), suggesting an excess of membrane vesicle material. This is supported by an apparent excess of membrane vesicles at weight ratios of 0.2 or greater as visualized by confocal microscopy (Fig. 4c, membranes labeled with DiO) or TEM (Fig. 4d). These representative images support the quantitative data in Fig. 4a, b. These data (Fig. 4a, b) also demonstrate that each component alone in suspension (“NP only” and “membrane only” conditions) exhibits significant increases in size and polydispersity over time, highlighting that both components can contribute to observed aggregation.

### Effect of suspension volume on coating efficiency

**Figure 5:**
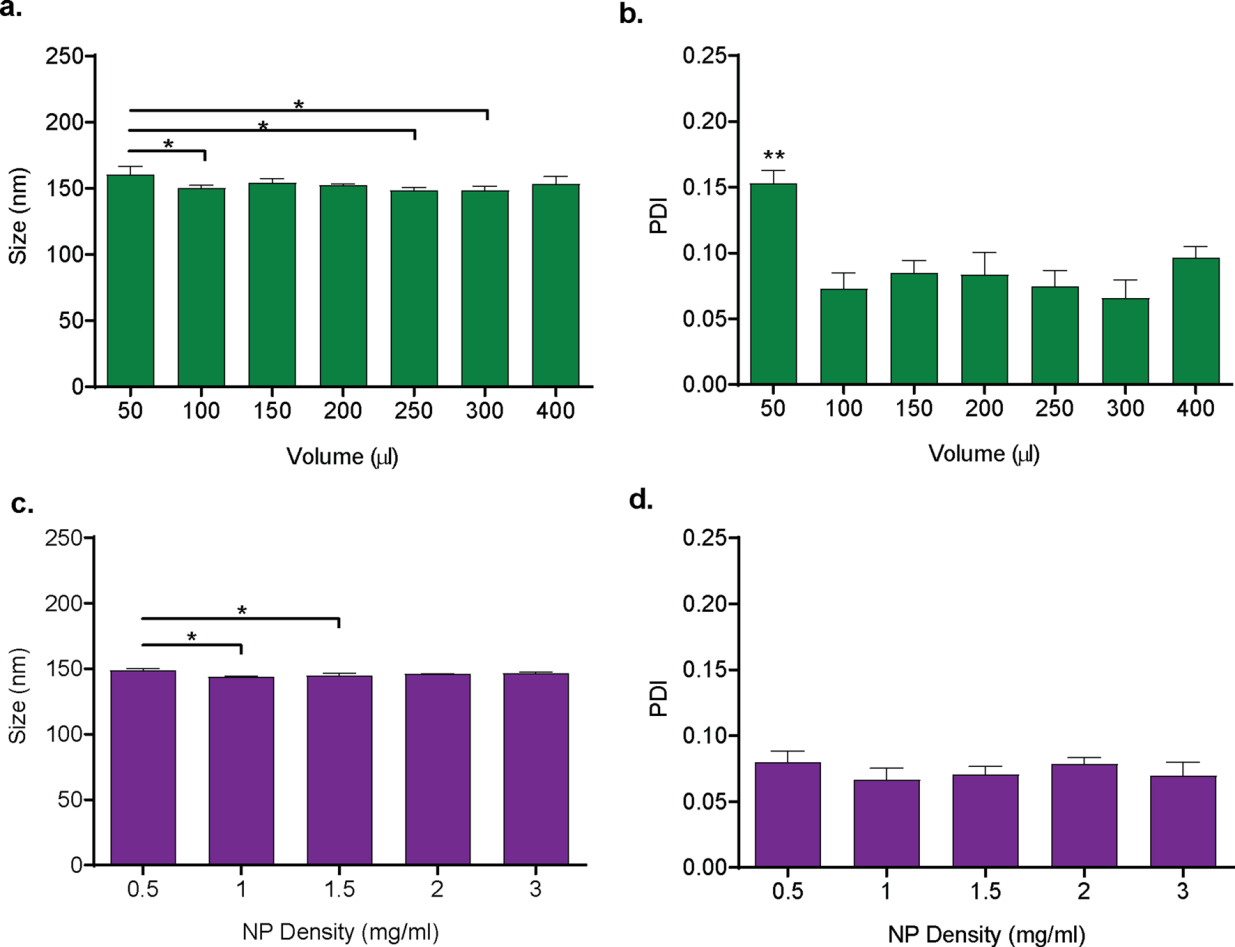
Effects of density and volume of the co-suspension on the efficiency of NP coating. PLGA nanoparticle (NP) and membrane vesicle co-suspensions at a ratio of 1.0 mg NP to 0.15 mg total membrane protein were sonicated (5 min., 15 °C, 60%) to induce coating and then incubated at room temperature under constant agitation for 4 days. (**a**) Size (nm) and (**b**) polydispersity index (PDI) of co-suspensions that were sonicated at different total volumes and a constant density of 1.0 mg/ml NPs. (**c**) Size (nm) and (**d**) PDI of co-suspensions that were sonicated at different NP densities, with a fixed volume of 150 µl. Data shown are all the mean ± SD, n = 3–4. * *p* < 0.05 as indicated, ** *p* < 0.05 vs. all other volumes, 1-way ANOVA with Tukey’s post hoc test.

To investigate the effect of sample volume on coating efficiency, co-suspensions of membrane vesicles and NPs at weight ratios of 0.1 were prepared in diH_2_O with different total volumes (50–400 ul) and sonicated at 20% amplitude, 5 min, 15 °C. After sonication, samples were diluted to a final NP concentration of 0.1 mg/mL in PBS and incubated at room temperature under constant agitation for seven days. Co-suspensions that were sonicated in a volume of 50 µl exhibited a small, but statistically significant, increase in size compared to groups sonicated in volumes of 100 µl, 250 µl, and 300 µl (Fig. 5a). The 50-µl sample was more polydisperse than all other groups (Fig. 5b). No significant difference in size or polydispersity was observed when samples were sonicated at volumes of 100–400 µl.

### Effect of NP density on coating efficiency

Coating of membrane vesicles onto the surface of PLGA NPs requires collision of the two components as a result of disruptive forces afforded by ultrasonic energy. We hypothesized that a lower density of the particles would decrease the chances of collision and reduce the coating efficiency. To investigate the effect of NP density on coating efficiency, we prepared co-suspensions of membrane vesicles and NPs in which the weight ratio of the components and the total volume were kept constant (0.1 and 150 µl, respectively) but the density of NPs varied (0.5–3 mg/ml). Co-suspensions were sonicated (20% amplitude, 5 min., 15 °C), diluted to a final NP concentration of 0.1 mg/mL in 1 × PBS, and incubated at room temperature under constant agitation for four days. Samples that were sonicated with a NP density of 0.5 mg/ml exhibited significantly larger size than samples that were sonicated with 1.0 or 1.5 mg/ml NP density (Fig. 5c). No significant difference in size was observed for samples that were sonicated with a NP density of 1–3 mg/ml, and no significant difference was observed in the polydispersity of all the groups (Fig. 5d).

### Optimization of sonication parameters by statistical design of experiments

Given the interdependence of the variables controlled by the sonicator and the ability of our quantitative outcomes to reveal small differences in coating efficiency, we chose to perform a multivariate statistical analysis by means of design of experiments (DoE), using Design-Expert software (Stat-Ease, Inc.). Three parameters (variables) were included in the statistical analysis (Table 1): amplitude (%, A), time (min., B), and temperature (°C, C). Membrane vesicles were combined with PLGA NPs (0.1 weight ratio) and sonicated according to the combinations of the levels of the variables listed in Supplementary Table S1. After sonication, samples were diluted in PBS and incubated under constant agitation for four days before quantitative assessment of size (nm) and PDI.

**Table 1:** Experimental range and levels of variables.

Variables	Units	Symbols	Levels				
			− 2	− 1	0	1	2
Amplitude	%	A	20	40	60	80	100
Time	Minutes	B	1	2	3	4	5
Temperature	C	C	5	10	15	20	25

The size and PDI of coated NPs were fit with a reduced cubic model and reduced 2FI model, respectively. Statistical significance of fit was determined by 1-way analysis of variance (ANOVA). For both size and PDI, the models are statistically significant (*p* < 0.001) and the lack of fits is not significant, indicating that the models are well fit (results in Supplementary Tables S2 and S3). For both response models, the differences between predicted R^2^ and adjusted R^2^ are < 0.2 with ratios > 4.0, indicating that both models can be used to navigate the design space. The final models for predicting the size and PDI are represented by Eqs. (1) and (2):

1 $$ \begin{aligned} \left( {\text{Size}} \right) & = 149.737 - 0.35A - 0.146324B + 3.64412C + 1.6875AB + 3.40313AC \\ & \,\,\,\, + 2.10625BC + 3.8451A^{2 } + 0.820099B^{2} + 2.07812ABC + 1.46875AB^{2} \\ \end{aligned} $$

2 $$ \begin{aligned} \left( {\text{PDI}} \right) & = 0.0912109 + 0.00919118A - 0.00794853B \\ & \,\,\,\,\, + 0.00205882C - 0.00846875AB - 0.00678125AC \\ \end{aligned} $$

Diagnostics were plotted to statistically validate our models (Fig. S3). For both size and PDI models, normal plots of residuals for NPs show run points following a straight line (Fig. S3A, B), indicating the residuals follow a normal distribution. Scatter plots of externally studentized residuals versus run numbers for both size and PDI (Fig. S3C, D) show random scatter, indicating no lurking variables that influenced the responses during the experiments. Plots of the predicted responses versus the actual (experimental) values (Fig. S3E, F) indicate that there are no groups of values that are not easily predicted by the models.

**Figure 6:**
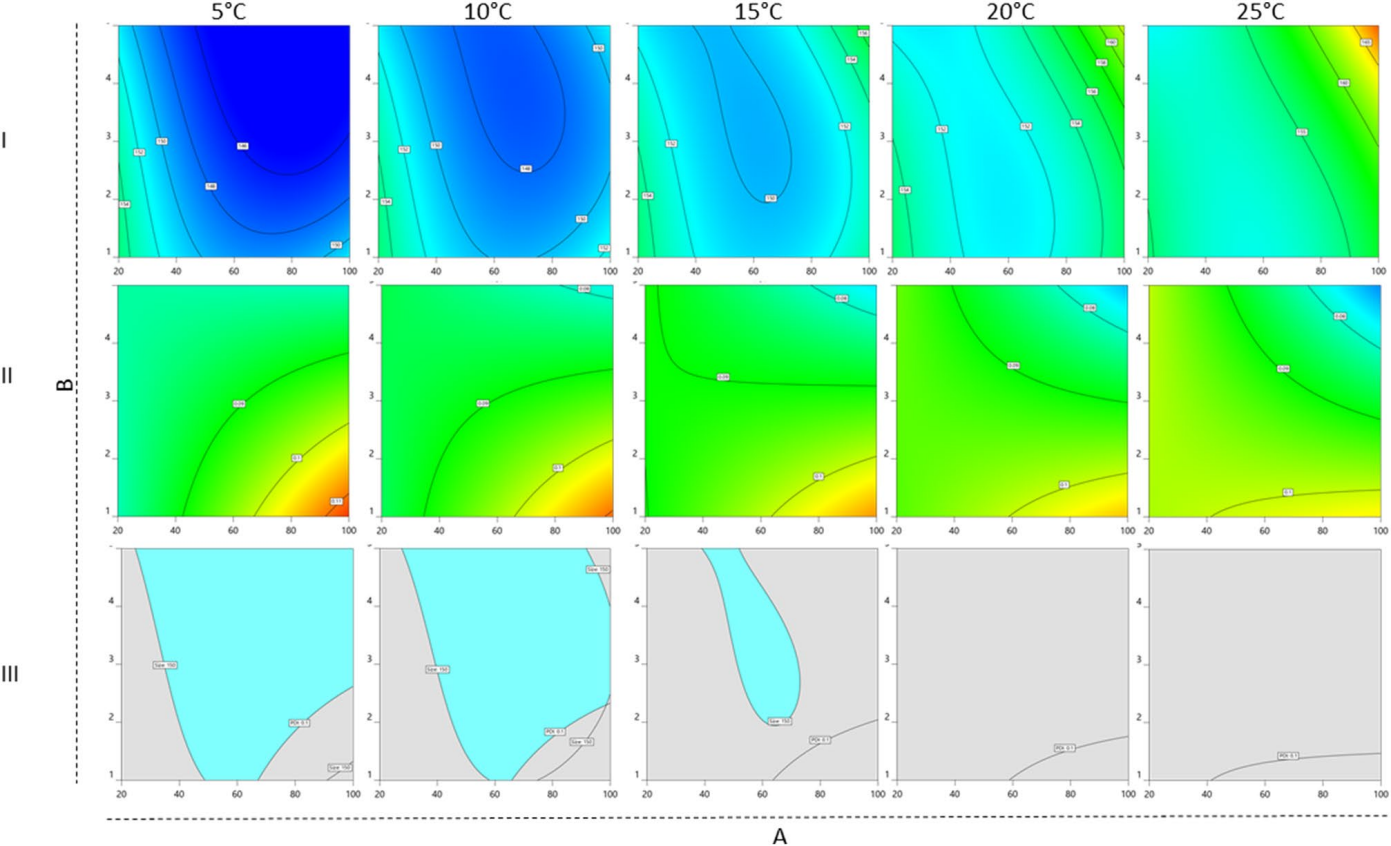
Contour plots generated in DesignExpert depicting A (amplitude, %, x-axis) versus B (duration, min., y-axis) at fixed C (temperature, °C, columns). Size (row I) and PDI (row II) responses at fixed variable C as indicated at the top of each column. Colors from warm to cool indicate values from high to low. Row III depicts overlay plots of the two responses where the criteria of size < 150 nm and PDI < 0.1 are fulfilled at each temperature.

**Figure 7:**
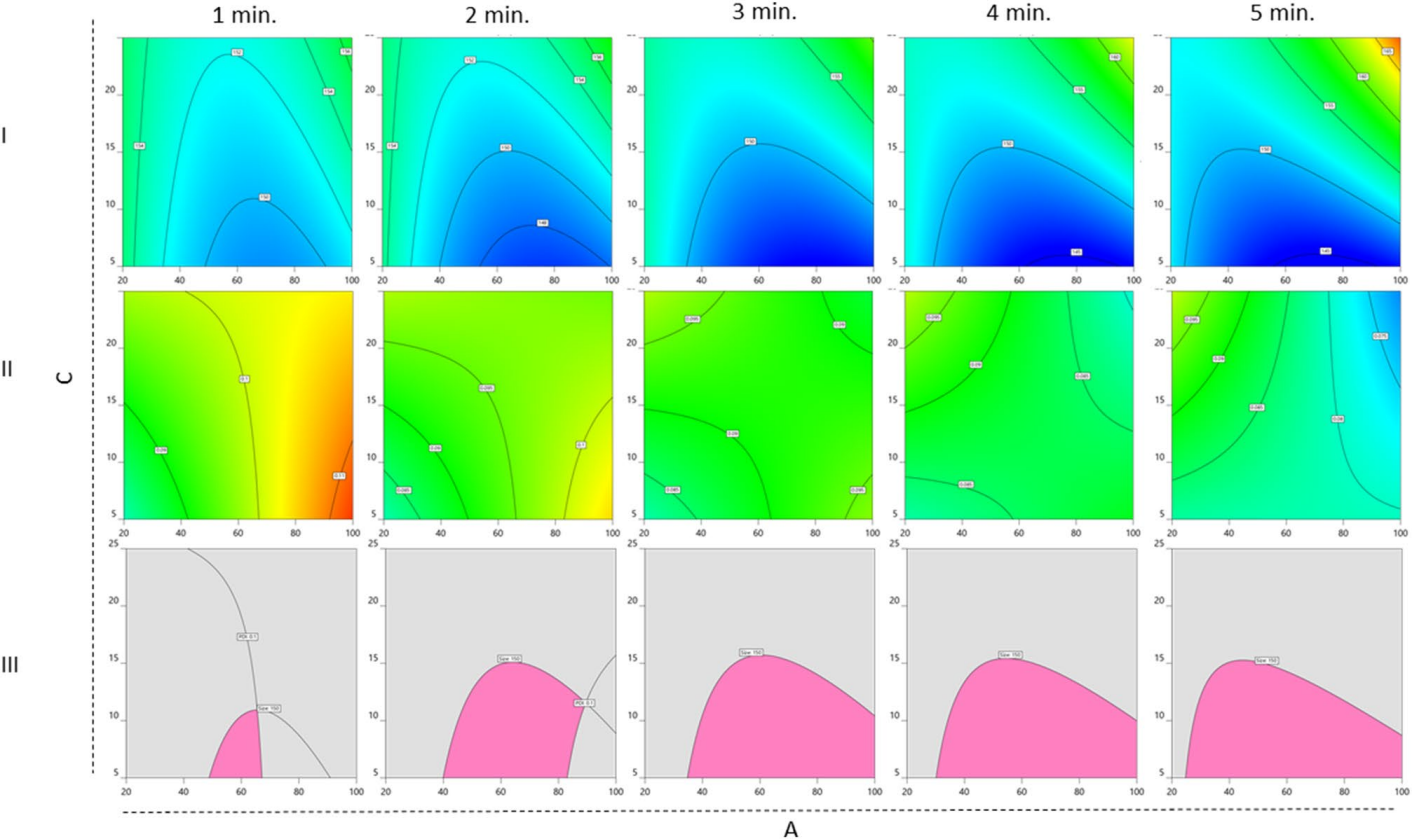
Contour plots generated in DesignExpert depicting A (amplitude, %, x-axis) versus C (temperature, °C, y-axis) at fixed B (duration, min., columns). Size (row I) and PDI (row II) responses at fixed variable C as indicated at the top of each column. Colors from warm to cool indicate values from high to low. Row III depicts overlay plots of the two responses where the criteria of size < 150 nm and PDI < 0.1 are fulfilled at each duration.

To successfully optimize sonication parameters, it is necessary to identify all interactions between variables, which can be visualized in contour plots (Figs. 6, 7). We utilized the optimization module of the software to identify suitable combinations of parameters with stringent selection criteria of the two responses: size < 150 nm and PDI < 0.1. Day 4 responses beyond those constraints suggest a trend towards aggregation and instability. Overlaid plots of the two responses assessed at different bath temperatures as a function of amplitude and time (Fig. 6, row III) reveal that at temperatures of 20 °C and above, there are no suitable combinations of amplitude and time that will produce coated NPs within the specified size and PDI selection criteria. Further, the area of the overlay decreases from 10 to 15 °C (Fig. 6, row III).

When investigating size and PDI at different durations as functions of amplitude and temperature (Fig. 7), the area of the overlay plots expands as the sonication time increases from 1 to 3 min (Fig. 7, row III). Beyond 3 min. of sonication this area remains the same, suggesting that 3 min. is sufficient time for coating (Fig. 7, row III).

## Discussion

Plasma membrane vesicles derived from an array of source cells can serve as a material for the surface functionalization of nanoparticles, facilitating both biomimetic cloaking and active targeting. Membrane proteins, retained by plasma membrane vesicles derived from source cells, can provide both a “marker-of-self” as well as a targeting moiety^1,26^. The hydrophilic surface imparted by the phospholipid bilayers enhances the colloidal stability of PLGA nanoparticles (NPs) in suspension and promotes increased circulation time. Methods for coating membrane vesicles onto NPs include sonication, extrusion, and microfluidic platforms^2^. Among these methods, sonication has been increasingly used due to its technical simplicity, its ability to both produce membrane vesicles from source cells as well as coat them onto cores, and the benefit of conservation of material over extrusion^17,27^. Despite its simplicity in execution, several variables influence the outcome of the process. In this work, we systematically investigated the effect of multiple parameters, including membrane to NP ratio, sample volume, density, sonication amplitude, duration, and temperature, on the outcomes of coating plasma membrane vesicles onto PLGA NP cores.

Bare PLGA nanoparticles are unstable as a colloidal suspension and aggregate non-uniformly in an ionic dispersant^28^^,^^22^, resulting in a significant increase in size and polydispersity^29^. Coating PLGA nanoparticles with PEG^9,30^, Poloxamer^31^, or with cell membrane^7,28,32^ yields a hydrophilic surface, imparts stability, and reduces the propensity to aggregate. While suspended in PBS and under constant agitation, the size and PDI of membrane-coated PLGA nanoparticles will only remain stable if the coating is complete^22^. In this study, we used increases in size and PDI as indicators of incomplete or insufficient membrane coating.

We coated membrane vesicles onto PLGA NP cores by sonicating co-suspensions of the two components. Sonication generates energy, and both PLGA NPs and membrane vesicles can exhibit changes in physical state in response to increased temperature^24,33^. Our sonicator was equipped with a recirculating chiller (Fig. S2) for temperature control, and initial experiments assessed the size and PDI of each component of our delivery platform after sonication at a range of temperatures. PLGA NPs significantly increased in size, but not PDI, when sonicated at 30 °C and 35 °C, indicating that NPs undergo glass transition and fuse at these temperatures (Fig. 2a, b). The glass transition temperature (*T*_g_) as a physical property of raw PLGA polymers under moisture-removing conditions is routinely reported; for PLGA 85:15, i.v. 0.55–0.75, *T*_g_ ranges from 50–55 °C (Lactel technical data). However, *T*_g_ of PLGA nanoformulations can be much lower^33^, particularly when in aqueous solution (32.1 °C) or encapsulated with increasing amounts of hydrophilic drugs (19.9–28.8 °C)^34^.

While initial experiments on membrane vesicles and NPs alone indicated that sonication at temperatures below 30 °C is sufficient to avoid phase transition and aggregation (Fig. 2), the importance of temperature control is further emphasized by our DoE contour plots. Overlays generated from A vs. B contour plots at a fixed temperature (Fig. 6) indicate that optimal criteria are not achievable after sonicating over 15 °C, and the area of the optimal “sweet spot,” or the range of variables A and B in which the size and PDI criteria are met, is greater at sonication at 5 °C or 10 °C. In other words, at lower temperatures, a wider range of amplitude and duration of sonication will result in a stably coated, monodisperse product. Our results indicate that the temperature of the water bath during sonication is an important variable to control and report.

Our multivariate analysis supports 3 min. of sonication as the amount of time needed for successful membrane coating of PLGA NPs. The area of the overlay plot decreases with less than 3 min. and remains constant between 3–5 min. As extended durations and higher amplitudes result in increased temperatures, sonicating for more than 5 min., particularly in the absence of temperature control, may lead to an unstable product. Based on our review of the literature, sonication is frequently performed for 2 or 3 min^11,28,35–39^. While longer durations have been reported when using non-polymeric core materials^40,41^, durations of 5 min.^42^ or 10 min.^43–45^ have also been reported for PLGA. Our data suggest that it may be difficult for other researchers to replicate positive results with longer sonication durations.

We investigated the influence of the membrane/PLGA NP ratio on the outcomes of size and PDI of the sonicated co-suspension. The ratio of the two components of the delivery system is a critical parameter to optimize. Separation of unconjugated excess material is problematic, with centrifugation resulting in fusion of or damage to membrane vesicles^46^, and filtration or exclusion chromatography resulting in a prohibitive loss of material that the use of sonication over extrusion attempts to circumvent. Either material in excess leads to aggregation and inaccurate estimation of drug delivered. Researchers have estimated the volume of blood required for complete, unilamellar coating of 80 nm PLGA cores^1^ and differently sized PLGA cores^22^ with erythrocyte membranes. In both examples, their empirical data support their theoretical calculations. The heterogeneity of membrane vesicles prepared from nucleated cells or from cell lines are prohibitive to accurate theoretical estimations of material needed, and empirical determination is necessary for identification of appropriate working ratios of vesicles and cores. As plasma membrane vesicles derived from different cell types are heterogeneous^47,48^, and NP core synthesis protocols vary between labs, this determination is best to be performed as needed.

Our multivariate analysis investigated the effects of and interactions between factors related to the energy delivered to the co-suspension. Co-suspensions with different compositions within the tube (sample volume, NP density) can respond differently to the same applied energy. Sample volume has been demonstrated to affect the efficiency of cell lysis and DNA shearing when disrupting cells with a bath sonicator for chromatin immunoprecipitation^49^. Sample density dictates the collision frequency of suspended particles^19^. Our data indicate that at low volume (50 µL) or low nanoparticle density (0.5 mg/mL), the sonicated co-suspensions exhibit small, but significant increases in size and PDI (Fig. 5). This demonstrates poor coating efficiency and suggests that the sonicated co-suspensions are unstable. We hypothesize that at extreme low volumes the delivered energy results in a more rapid temperature increase. At extreme low densities, the probability of collision with another suspended particle or vesicle is too low. Complete characterization of this effect is beyond the scope of this work. Our data suggest that a volume of 100–400 ul and NP density of 1–3 mg/ml are appropriate working ranges, and that controlling for these parameters between separate batches or experiments is critical.

Membrane coating of PLGA NPs with cell-derived membrane vesicles is a strategy for targeted drug delivery that has been increasingly used for a diverse array of applications. As more research groups undertake this method, it is important to consider factors that affect experimental reproducibility so that results can be interpreted in the context of hypothesis-testing and not just method validating. Here, we investigated the relationship between variables that influence the energy delivered to co-suspensions of PLGA NPs and membrane vesicles. We measured success by the stability of sonicated co-suspensions in PBS, as membrane coating reduces the degree of non-uniform aggregation of PLGA NPs. Although this work does not provide a standardized protocol and conditions, the identification of interactions between parameters involved in membrane coating with sonication can assist other researchers in developing a reproducible protocol for coating drug-loaded PLGA cores with membrane vesicles from different source cells.

## Materials and methods

### Materials

Ester-terminated poly(lactic-co-glycolic)acid (PLGA 85:15, viscosity 0.55—0.75) was obtained from Durect corp (Cupertino, CA). Dichloromethane (DCM), acetonitrile (MeCN), dimethyl sulfoxide (DMSO), poly(vinyl alcohol) (PVA, MW 31,000–50,000, 87–89% hydrolyzed), dithiothreitol, trypsin-chymotrypsin inhibitor and phenylmethylsulfonyl fluoride (PMSF), as well as all other reagents, unless noted otherwise, were purchased from Millipore Sigma (St. Louis, MO). 1,1'-dioctadecyl-3,3,3',3'-tetramethylindodicarbocyanine, 4-chlorobenzenesulfonate salt (DiD) and CellMask™ Orange Plasma Membrane Stain were purchased from Thermo Fisher Scientific (Waltham, MA). The bath sonication system was composed of Fisherbrand Model 505 Sonic Dismembrator, a cup horn (Qsonica LLC, Newtown, CT), and a recirculating chiller (Qsonica LLC) (Fig. S2A). The cup horn is equipped with a tube holder that can hold eight 1.5 ml tubes (Fig. S2B).

The following primary antibodies were obtained commercially and used at the indicated dilutions: 1:1,000 rabbit anti-COX IV and 1:1,000 rabbit anti-GAPDH conjugated with horseradish peroxidase (Cell Signaling Technology, Danvers, MA); 1:1,000 rabbit anti-CD45 (Thermo Fisher Scientific); 1:1,000 rabbit anti-CD11a, 1:1,000 rabbit anti-nucleoporin p62, and 1:10,000 rabbit anti-sodium potassium ATPase (Abcam, Cambridge, MA). Horseradish peroxidase-conjugated AffiniPure goat anti-rabbit IgG (H + L) secondary antibody (1:10,000) was purchased from Jackson ImmunoResearch Laboratories (West Grove, PA).

### Cell culture

Jurkat T lymphocyte cell line was purchased from American Type Tissue Collection (Manassas, VA) and maintained in suspension in RPMI 1640 (ATCC) supplemented with 10% fetal bovine serum (FBS, Thermo Fisher Scientific), 100 U/ml penicillin–streptomycin, and 50 ug/ml amphotericin B at a density of 10^4^ to 10^5^ cells/ml.

### Isolation of subcellular membrane fractions

Plasma membranes were extracted from Jurkat cells. Briefly, 2.8 × 10^8^ cells were collected by centrifugation at 700 g for 5 min and resuspended in *homogenization buffer* containing sucrose (250 mM), Tris hydrochloride (10 mM), magnesium chloride (1 mM), potassium chloride (1 mM), PMSF (2 mM), trypsin-chymotrypsin inhibitor (200 μg/mL), deoxyribonuclease (10 μg/mL), and ribonuclease (10 μg/mL) for 30 min. The cell suspension was gently homogenized using a Dounce homogenizer and laid on top of a sucrose density gradient (30%, 40%, 55% w/v). Subcellular fractions were separated by ultracentrifugation with a Beckman SW-28 rotor at 28,000 g for 45 min at 4 °C. Three lipid-rich fractions at the sucrose interfaces were collected, diluted 1:1 with 150 mM saline, and pelleted by centrifugation at 28,000 g for 60 min. The pellets were lyophilized and stored at − 80 °C until use.

### Western blot

Lyophilized fractions were rehydrated with diH_2_O supplemented with protease inhibitor cocktail (Roche, Basel, Switzerland), and protein concentration was assessed using the Pierce Rapid Gold BCA Protein Assay Kit (Thermo Fisher Scientific) according to the manufacturer’s protocol. Samples of membrane lysates (25 µg protein) were prepared with 4 × Laemmli sample buffer (Bio-Rad, Hercules CA) and dithiothreitol (100 mM), heated to 95 °C for 5 min, and resolved on a Mini-PROTEAN® TGX™ Precast Gel (Bio-Rad) by sodium dodecyl sulfate–polyacrylamide gel electrophoresis. Proteins were transferred onto nitrocellulose membranes, blocked with 5% non-fat dry milk in tris-buffered saline containing 0.1% Tween-20 (30 min., 37 °C), probed with the indicated primary and corresponding secondary antibodies, and detected using SuperSignal West Pico PLUS Chemiluminescent Substrate (Thermo Fisher Scientific). Blots were imaged and processed using the ChemiDocTM XRS + with Image LabTM software (Bio-Rad).

### Nanoparticle synthesis

Nanoparticles (NPs) were prepared by oil-in-water single emulsion^50^. Poly (lactic-co-glycolic) acid (PLGA) (100 mg, 85:15, inherent viscosity 0.55–0.75, Durect Corp.) was dissolved in 1 ml organic solvent (DCM and MeCN, 1:1 (v/v)) and added dropwise to 6 ml of 6% PVA in diH_2_O, w/v) under vigorous vortexing. The mixture was sonicated using an ultrasonic processor with a micro-tip (Cole-Parmer, Vernon Hills, IL) for 10 rounds of 30 s on and 30 s off while in an ice bath. After sonication, the emulsion was added to a beaker containing 100 ml diH_2_O and stirred overnight at 300 rpm to allow the organic solvent to evaporate. The NPs were collected by centrifugation at 40,000 g for 30 min and washed three times with diH_2_O. After the final wash, NPs were resuspended in diH_2_O containing 50 mg sucrose, frozen at  − 80 °C, lyophilized overnight, and stored at  − 20 °C in a desiccator. For fluorescent labeling, DiD was added to the organic phase (0.3% w/w with PLGA).

### Nanoparticle characterization

Size, polydispersity index (PDI), and zeta potential of NPs were assessed with dynamic light scattering using a Zetasizer Nano ZS90 (Malvern Panalytical, Westborough, MA). A suspension of lyophilized NPs (0.1 mg/ml) was prepared in 0.2 µm-filtered diH_2_O and transferred to a disposable polystyrene cuvette or capillary cell. For size measurement, the dispersant was set to diH_2_O (η = 0.8872 cP, *n* = 1.33) at 25 °C. NPs were equilibrated for 3 min. in the cuvette and measured at a 90° angle. Each measurement was an average of four separate, consecutive measurements performed by the Zetasizer that met 12 indicators for result quality criteria. The same dispersant specifications were utilized for zeta potential measurement as a function of electrophoretic mobility, plus a dielectric constant of 78.5. The zeta measurements were based on F(ka) model using Henry’s function. NPs were equilibrated for 1 min in the cuvette and measured as described above.

### Membrane coating of PLGA NP cores

Prior to use as a coating material, reconstituted membrane vesicles were processed by a series of centrifugation and sonication steps. Lyophilized membrane fractions were reconstituted in 1 ml of chilled diH_2_O, vortexed, and centrifuged (16,000 g, 30 min., 4 °C). The pellet was resuspended in diH_2_O as above and sonicated at 100% amplitude (5 min., 4 °C). The sonicator exhibits a fixed output frequency (20 kHz) and a maximum power of 500 watts (Fig. S2). A recirculating chiller controls the temperature of the bath (Fig. S2). The sample was subject to another round of centrifugation and sonication as above to remove residual salt and reduce vesicle size. The protein concentration of the final vesicle suspension was measured using Pierce Rapid Gold BCA Protein Assay Kit. Size and PDI were assessed with dynamic light scattering as described above on a 0.1 mg/ml by protein weight suspension of membrane vesicles in diH_2_O.

Processed plasma membrane vesicles that met size and PDI criteria were coated onto prepared PLGA NP cores by sonication. The general protocol for coating was to combine a predetermined amount of membrane vesicles (protein weight) and PLGA NP cores (dry weight) in a volume of diH_2_O in a 1.5 ml polypropylene tube and sonicate the tube at a predetermined amplitude (%), temperature (°C), and duration (min.). In some experiments, size and PDI of the suspension were measured immediately after coating. In other experiments, the suspension was diluted to a final concentration of 0.1 mg/ml NPs in 1 × PBS and incubated at room temperature under constant agitation in the form of 360° inversion on a HulaMixer (Thermo Fisher Scientific) at 8 rpm to induce aggregation prior to measuring size and PDI.

### Experimental workflow and design of experiments

Initial experiments investigated the effects of water bath temperature (5 °C-35°C) on the size and PDI of each of the two components, NPs and membrane vesicles, sonicated alone in suspension (100% amplitude, 5 min.). Next, we validated the quantitative outcomes of our experimental design- increases in size and PDI- as indicators of instability and incomplete coating. Per the general protocol for coating membrane vesicles onto NP cores described above, NPs (0.5 mg/ml) were mixed with membrane vesicles (0.05 mg/ml) in diH_2_O and sonicated at 60% amplitude, 15 °C, for 5 min. After sonication, samples were diluted to a final NP concentration of 0.1 mg/ml in 1 × PBS and incubated at room temperature under constant agitation. Parallel control samples were either not sonicated or not agitated.

We then used the general protocol and sonicated different membrane to NP weight ratios (ranging from NP only to 1.0) of the two components in co-suspension at a fixed amplitude (20%), temperature (15 °C), and duration (5 min.). Using the optimal ratio identified in these experiments, we assessed size and PDI of co-suspensions that were sonicated at different volumes (50–400 µl) and NP densities (0.5–3 mg/ml).

Finally, to identify effects of and interactions between the settings on the sonicator, we performed statistical design of experiments (DOE) with Design-Expert software (Stat-Ease, Version 13, Minneapolis, MN) with a 5-level surface central composite design algorithm. Three parameters (variables) were included in the statistical analysis (Table 1): amplitude (%, A), time (min., B), and temperature (°C, C). These variables were incorporated into the development of a Central Composite design- a Response Surface Method that evaluates interactions and quadratic effects by analyzing the variables at five levels. The 32 run combinations used to test the model are listed in Supplementary Table S1. PLGA NPs (1 mg/ml final concentration) were combined with membrane vesicles (0.1 mg/ml final protein concentration) in a volume of 150 µl in 1.5 mL polypropylene tubes. Co-suspensions were sonicated according to the conditions outlined in Supplementary Table S1. After sonication, samples were diluted in PBS and incubated as described above before quantitative assessment of size (nm) and PDI. The evaluated responses (raw values in Supplementary Table S1) were analyzed by ANOVA, allowing us to assess the effect of each variable and their interactions.

### Imaging

We used transmission electron microscopy (TEM) and confocal microscopy to visualize morphology and colocalization, respectively, of membrane vesicle and NP components after sonicating different ratios of the two as described above. For TEM imaging, carbon-coated 200 mesh copper grids (Electron Microscopy Sciences, Hatfield, PA) pretreated with 0.002% Alcian blue in 0.03% acetic acid were floated on top of 30 µl drops of NP suspensions (30 min, room temperature). After washing with diH2O, the samples were negatively stained by floating the grid on a 50 µl drop of filtered 2% uranyl acetate (pH = 7.0, 5 min, room temperature). Samples were dried for 12 h in a grid storage box before imaging with a Philips CM120 transmission electron microscope (TSS Microscopy, Beaverton, OR).

For confocal microscopy, NPs loaded with lipophilic tracer DiD (0.3% w/w) were suspended with varying amounts of membrane vesicles labeled with CellMask™ orange plasma membrane stain and sonicated as described above. Commercial cyanines are routinely used to label PLGA NPs^51^, membrane vesicles including liposomes and exosomes^52^, and each component of membrane coated PLGA NPs without interfering with the properties of the components or the system^53^. Samples were mixed with an aqueous mounting medium and mounted on glass slides with 12 mm glass coverslips (#1.5H thickness, ThorLabs, Newton, NJ). Slides were imaged using 561 nm wavelength (membrane vesicles, green) and 633 nm wavelength (NP, red) using Leica HCS A confocal microscope.

#### Statistics

One-way or two-way repeated-measures ANOVA, followed with Tukey’s or Dunnett’s post hoc analysis, were used for comparison of data groups, where appropriate and as indicated in figure legends. In all cases, *p* < 0.05 was considered statistically significant.

## Supplementary Information

Supplementary Information.
