# Supplementary material for: Optimization of critical parameters for coating of polymeric nanoparticles with plasma membrane vesicles by sonication
Source: Sci Rep. 2021 Dec 14;11:23996. doi: 10.1038/s41598-021-03422-5 (PMC8671476; doi:10.1038/s41598-021-03422-5)
Supplement: Supplementary file 1 — Supplementary Information. [file 41598_2021_3422_MOESM1_ESM.docx]

**Optimization of critical parameters for coating of polymeric nanoparticles with plasma membrane vesicles by sonication**

Feipeng Yang, ^1,2^ Maleen H. Cabe, ^1,2^ Sean D. Ogle, ^1^ Veronica Sanchez,^2^ and Kelly A. Langert ^1,2^

Department of Molecular Pharmacology and Neuroscience

Loyola University Chicago, Stritch School of Medicine, Maywood, IL 60153 (U.S.A.) ^1^

Research Service

Edward Hines Jr. VA Hospital, Hines, IL 60141 (U.S.A.) ^2^

Figure S1. Uncropped western immunoblots.

Figure S2. PLGA nanoparticle surface coating with membrane vesicles through bath sonication.

Figure S3. Diagnostics of the DOE model.

Figure S4. Confocal and TEM images of PLGA nanoparticles and membrane vesicles.

Table S1. Report of actual and predicted response from the surface central composite design.

Table S2. Analysis of variance (ANOVA) results for sizes of the coated nanoparticles.

Table S3. Analysis of variance (ANOVA) results for PDI of the coated nanoparticles.


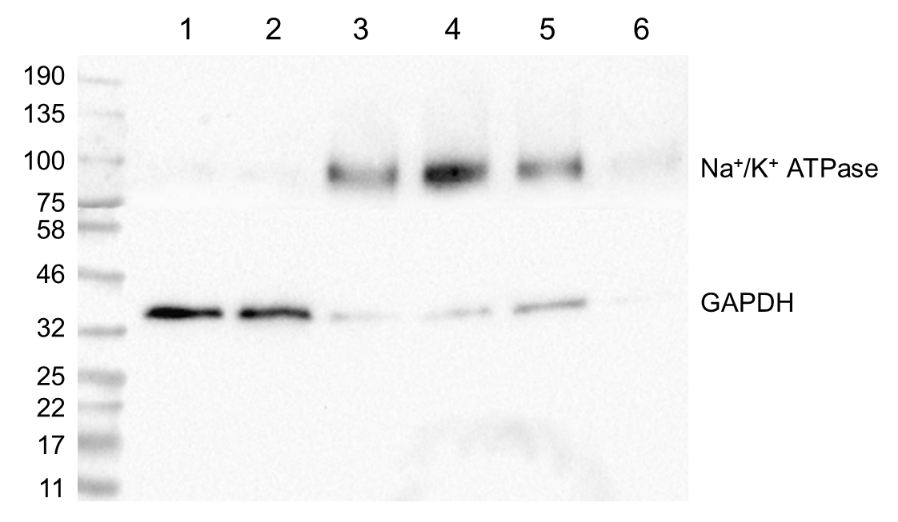


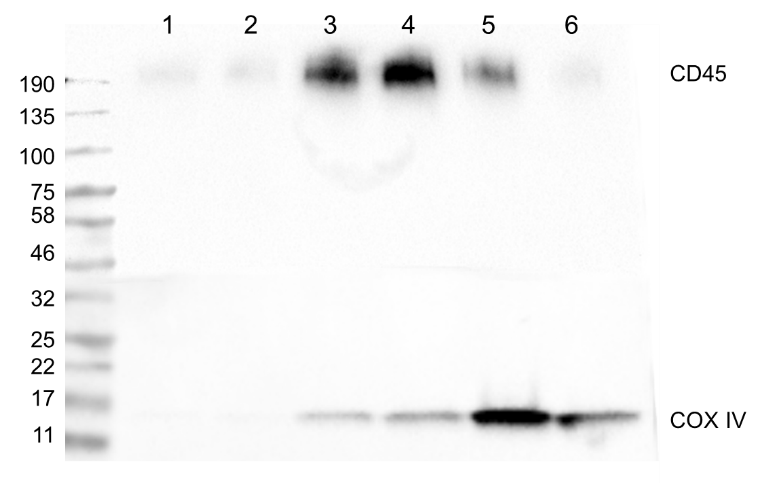


**
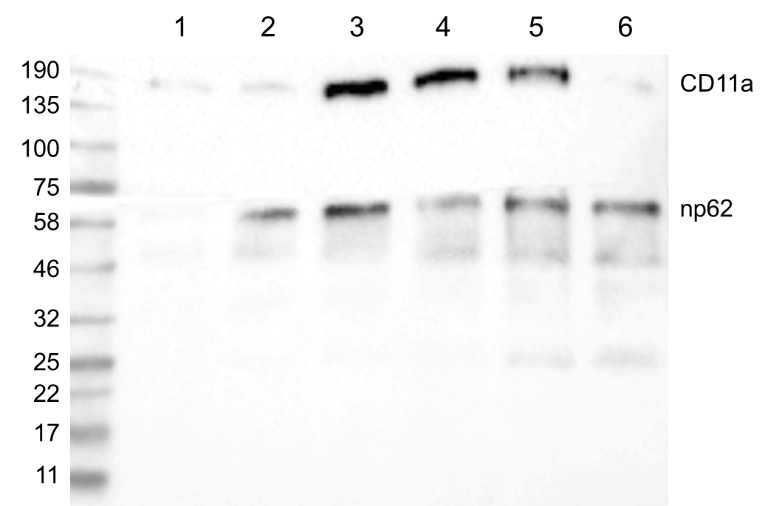
**

**Figure S1.** Full length Western blots depicted in Fig. 1 of the main text. Proteins in six subcellular fractions were separated onto three nitrocellulose membranes, and each membrane was cut and probed with two primary antibodies as indicated.


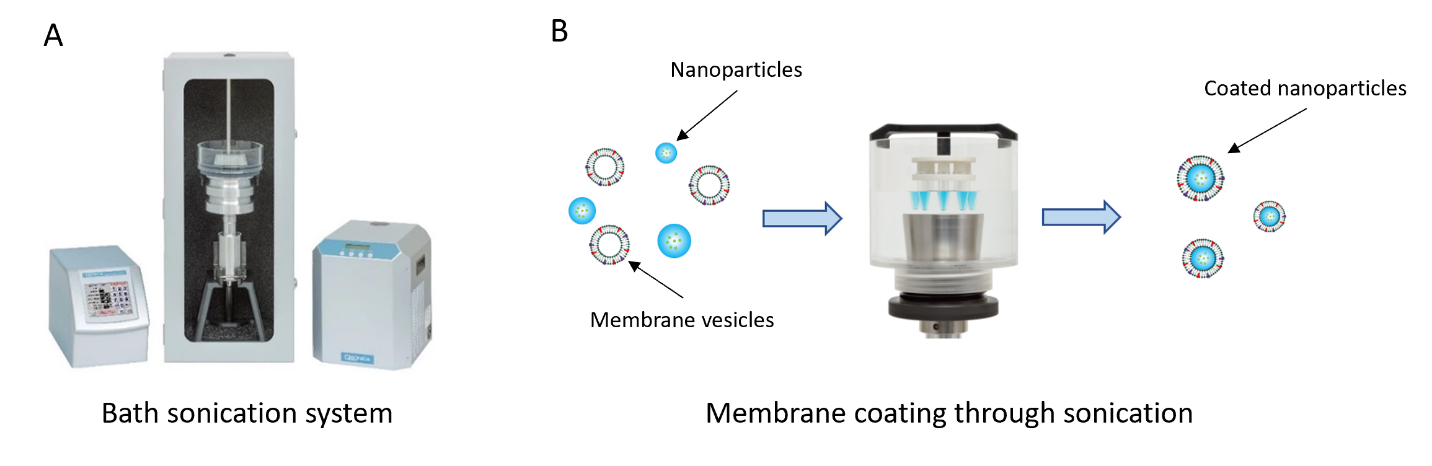


**Figure S2.** PLGA nanoparticle surface coating with membrane vesicles through bath sonication. **(A)** Bath sonication system composed of Fisherbrand Model 505 Sonic Dismembrator, a cup horn (Qsonica, LLC, #431C2), and a recirculating chiller equipped with real-time temperature read out (Qsonica, LLC, #4905). **(B)** Illustration of membrane coating process through bath sonication.


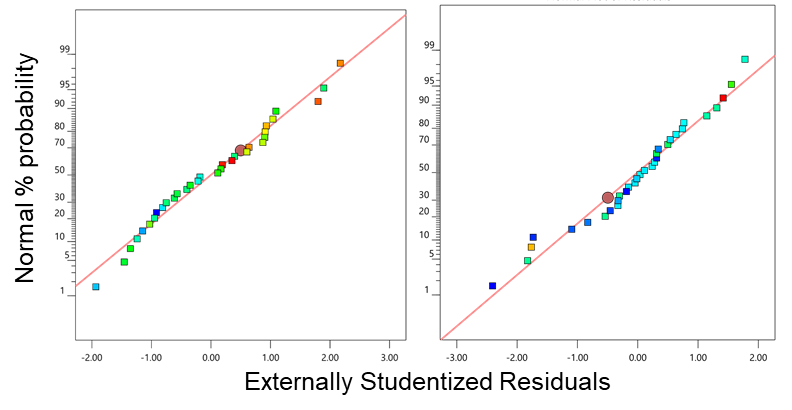


**
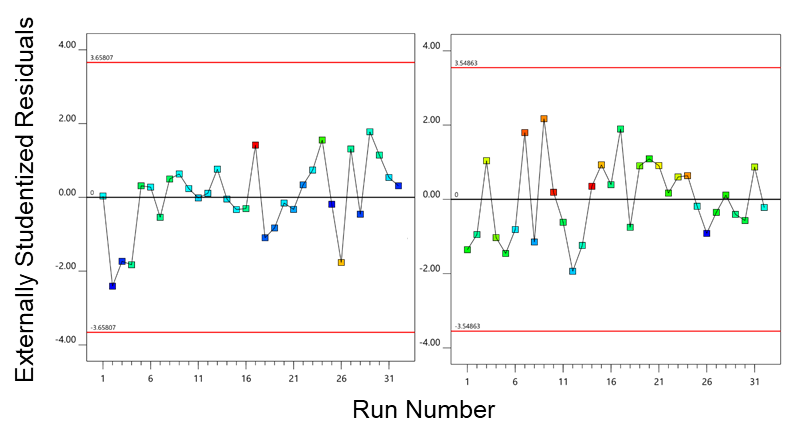
**

**
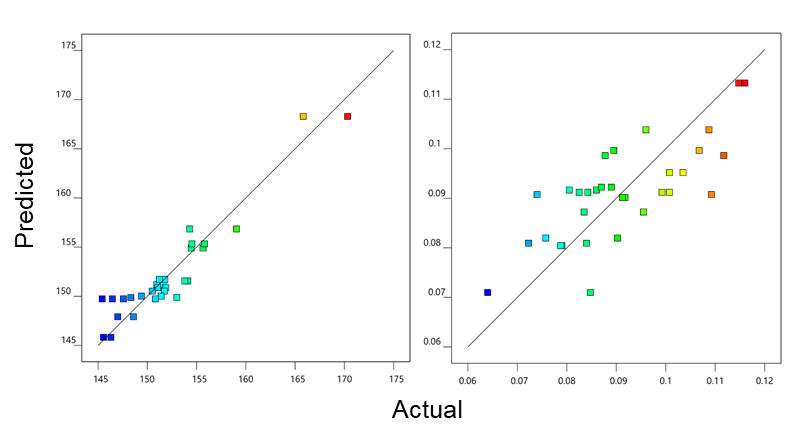
**

**Figure S3.** DOE diagnostics. Normal plots of residuals for NP size **(A)** and PDI **(B)**. Scatter plots of externally studentized residuals versus run number for size **(C)** and PDI **(D)**. Scatter plots of predicted value versus actual value from the established models for size **(E)** and PDI **(F)**.


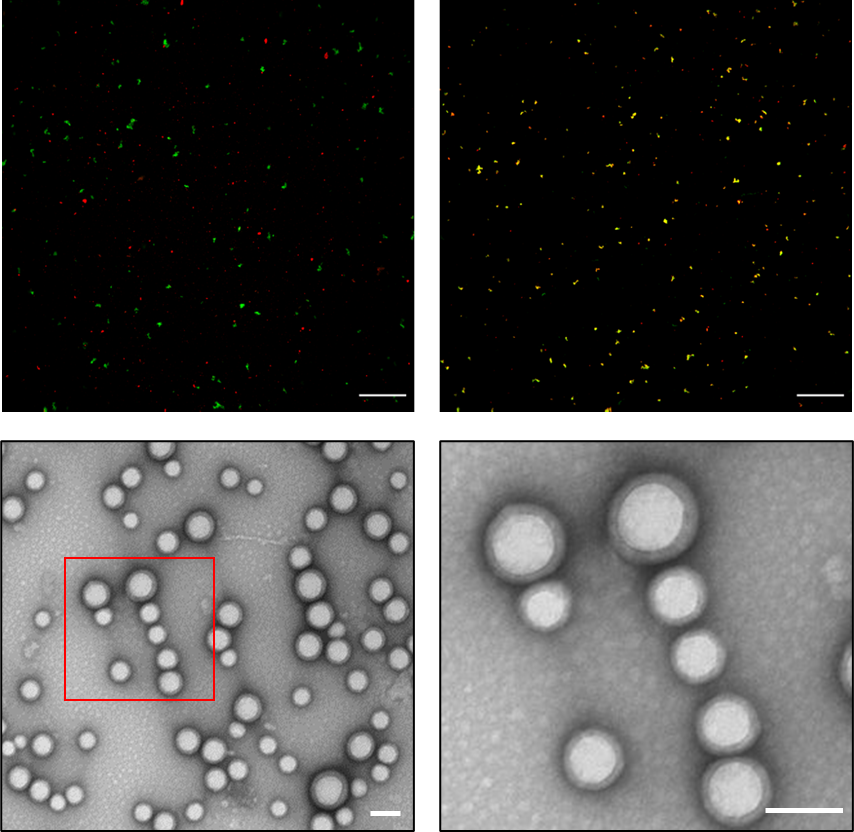


C.

D.

B.

A.

**Figure S4.** Confocal and TEM images of PLGA NPs and membrane vesicles. Co-suspensions of PLGA NPs (red) and membrane vesicles (green) before **(A)** and after **(B, C, D)** bath sonication. D is the inset indicated in C. Confocal scale bar: 20 µm; TEM scale bar: 200 nm.

Table S1. Report of actual and predicted response from the surface central composite design.

| **Variables** | | | | **Response** | | | | | |
| --- | --- | --- | --- | --- | --- | --- | --- | --- | --- |
|  |  |  |  | **Size(nm)** | | | | **PDI** | |
| **Run** | **A** | **B** | **C** | | **Actual** | **Predicted** | **Actual** | | **Predicted** |
| 1 | 20 | 5 | 25 | | 151.8 | 151.7 | 0.090 | | 0.100 |
| 2 | 60 | 3 | 15 | | 145.4 | 149.7 | 0.083 | | 0.091 |
| 3 | 60 | 3 | 15 | | 146.5 | 149.7 | 0.101 | | 0.091 |
| 4 | 100 | 1 | 25 | | 154.3 | 156.8 | 0.096 | | 0.104 |
| 5 | 20 | 1 | 25 | | 155.8 | 155.3 | 0.088 | | 0.099 |
| 6 | 20 | 5 | 5 | | 151.6 | 151.2 | 0.076 | | 0.082 |
| 7 | 20 | 1 | 25 | | 154.5 | 155.3 | 0.112 | | 0.099 |
| 8 | 20 | 1 | 5 | | 155.7 | 154.9 | 0.072 | | 0.081 |
| 9 | 40 | 3 | 15 | | 151.9 | 150.9 | 0.109 | | 0.091 |
| 10 | 100 | 1 | 5 | | 151.5 | 151.1 | 0.115 | | 0.113 |
| 11 | 80 | 3 | 15 | | 150.5 | 150.5 | 0.086 | | 0.092 |
| 12 | 40 | 3 | 15 | | 151.1 | 150.9 | 0.074 | | 0.091 |
| 13 | 80 | 3 | 15 | | 151.7 | 150.5 | 0.081 | | 0.092 |
| 14 | 100 | 1 | 5 | | 151.1 | 151.1 | 0.116 | | 0.113 |
| 15 | 20 | 5 | 25 | | 151.2 | 151.7 | 0.107 | | 0.100 |
| 16 | 20 | 1 | 5 | | 154.5 | 154.9 | 0.084 | | 0.081 |
| 17 | 100 | 5 | 25 | | 170.3 | 168.3 | 0.085 | | 0.071 |
| 18 | 60 | 3 | 15 | | 147.6 | 149.7 | 0.084 | | 0.091 |
| 19 | 60 | 4 | 15 | | 148.3 | 149.9 | 0.0096 | | 0.087 |
| 20 | 20 | 5 | 5 | | 151. | 151.2 | 0.090 | | 0.082 |
| 21 | 60 | 2 | 15 | | 149.4 | 150.0 | 0.104 | | 0.095 |
| 22 | 60 | 3 | 10 | | 148.6 | 147.9 | 0.092 | | 0.090 |
| 23 | 60 | 2 | 15 | | 151.4 | 150.0 | 0.101 | | 0.095 |
| 24 | 100 | 1 | 25 | | 159.1 | 156.8 | 0.109 | | 0.104 |
| 25 | 100 | 5 | 5 | | 145.6 | 145.8 | 0.079 | | 0.080 |
| 26 | 100 | 5 | 25 | | 165.8 | 168.3 | 0.064 | | 0.071 |
| 27 | 60 | 3 | 20 | | 154.1 | 151.6 | 0.089 | | 0.092 |
| 28 | 60 | 3 | 10 | | 147.0 | 147.9 | 0.091 | | 0.090 |
| 29 | 60 | 4 | 15 | | 153.0 | 149.9 | 0.084 | | 0.087 |
| 30 | 60 | 3 | 20 | | 153.8 | 151.6 | 0.087 | | 0.092 |
| 31 | 60 | 3 | 15 | | 150.8 | 149.7 | 0.099 | | 0.091 |
| 32 | 100 | 5 | 5 | | 146.3 | 145.8 | 0.079 | | 0.080 |

Table S2. Analysis of variance (ANOVA) results for sizes of the coated nanoparticles.

Table S3. Analysis of variance (ANOVA) results for PDI of the coated nanoparticles.
